# Respiratory Distress in Neonate With Scimitar Syndrome

**DOI:** 10.7759/cureus.12299

**Published:** 2020-12-26

**Authors:** Nabil Al Qasimi, Ahmed M Al Qassimi

**Affiliations:** 1 Pediatric Cardiology, Maternity and Children’s Hospital-Dammam, Dammam, SAU; 2 Pediatrics, King Faisal University, Dammam, SAU

**Keywords:** scimitar syndrome, respiratory distress, dextroposition of the heart, dyspnea

## Abstract

Scimitar syndrome is a relatively uncommon association of congenital cardiopulmonary anomalies characterized by partial or complete anomalous pulmonary venous drainage from the right lung. It can present in the neonatal period, as well as later in life. In this paper, we present a case of a 15-day-old Saudi boy diagnosed with scimitar syndrome who presented with a chest infection and respiratory distress, along with a brief review of the literature. Key diagnoses, such as chest x-ray, echocardiography, and computerized tomography of the chest, led to the conclusion that the patient was a rare scimitar syndrome case. Understanding that scimitar syndrome usually presents with variable symptoms, we had to perform several diagnostic tests before concluding this was a scimitar syndrome case. Infantile scimitar syndrome requires a high degree of suspicion for early referral and management, so we needed to conduct a special diagnostic test. Our literature review of scimitar syndrome has supported our findings and encouraged us to share this rare scimitar syndrome case.

## Introduction

This report presents a case of a newborn encountered at our centre who had an early presentation of respiratory distress and lower respiratory tract infection diagnosed as scimitar syndrome. This report aims to review the clinical presentations and radiological findings of the case, with a literature review.

To help fully understand and diagnose the case, we provide a thorough literature review. As per the literature, scimitar syndrome (also called hypogenetic lung syndrome, Halasz syndrome, and congenital pulmonary venolobar syndrome) is a rare variant of partial anomalous pulmonary venous return with an aberrant vein. In these cases, the scimitar vein drains the right lung to the inferior vena cava (IVC) instead of the left atrium, resulting in a left-to-right shunt. The classic frontal radiographic finding, designated as "the scimitar sign," is of a density shaped like a scimitar (a curved Turkish sword) along the right cardiac border [1-3]. Overall, our case well matched this definition.

Scimitar syndrome is named after the curvilinear vascular pattern created on a chest radiogram by the anomalous pulmonary vein coursing along the heart's right side towards the diaphragm [3]. Lung radiography can show this characteristic appearance, but this typical radiological sign is not seen in most cases, similar to our case. As a result, further imaging, such as magnetic resonance angiography or computerized tomography (CT), is needed to confirm the diagnosis [4]. In our case, we performed a CT scan to confirm the findings and arrive at a proper diagnosis.

Other features of the syndrome include right lung hypoplasia, dextroposition of the heart, right pulmonary artery hypoplasia, pulmonary sequestration, atrial septal defect (ASD), and systemic collaterals from the aorta [1,2,5]. Symptoms can start during infancy or beyond (childhood/adult form). The syndrome has a variable presentation, ranging from asymptomatic infants to those with heart failure and severe pulmonary hypertension. The infantile form presents early in life, with tachypnea symptoms, repeated chest infection, heart failure, cyanosis, and failure to thrive.

Among patients without significant associated congenital heart disease, those in the "isolated infantile" group often have significant pulmonary hypertension and present with heart failure before one year of age [6]. By contrast, those in the milder "isolated adult" group have symptoms, if any, that occur years to decades after birth [7]. The triad of respiratory distress, right lung hypoplasia, and dextroposition of the heart should alert the clinician to the possibility of this syndrome [1].

The literature also indicates that this syndrome's prevalence is very rare, accounting for 1 to 3 per 100,000 live births [7], with a female/male ratio of 2/1 [8]. The true incidence may be higher because many patients are asymptomatic. The significant complications depend on the age of onset and the severity of the clinical symptoms. The most crucial scimitar syndrome complications are recurrent pulmonary infections, congestive heart failure, and pulmonary hypertension [8,9].

Few published studies have focused only on the infantile type of scimitar syndrome, especially in Saudi Arabia. Most of the literature reports are limited to case reports and a few case series. To our knowledge, the occurrence of infantile scimitar syndrome has not been fully reported in the pediatric literature in Saudi Arabia.

## Case presentation

A 15-day-old Saudi male child was born at term to a gravida six mother by normal vaginal delivery. The pregnancy period was uncomplicated. The infant's birth weight was 3.5kg, and Apgar scores were 8 and 9 at one and five minutes, respectively. The patient presented to the emergency room with tachypnea and respiratory distress and was admitted to the nursery as a case of chest infection.

He weighed 3.5kg (25th percentile) on physical examination, his height was 52 cm (50th percentile), and his head circumference was within normal limits. He showed a respiratory rate of 62 breaths/min, peak heart rate of 161 beats/min, blood pressure of 88/43 mm/Hg, and a body temperature of 36.8°C. A respiratory system examination and auscultation of both lungs revealed marked rales at the basal pulmonary segments. A cardiovascular examination revealed bilaterally palpable peripheral pulses and normal capillary filling time. The S1, S2, and sounds were normal, but a grade II/VI systolic murmur was detected at the left lower sternal border, not radiating and with no thrill or parasternal heave. Examination of other systems was unremarkable.

**Figure 1:**
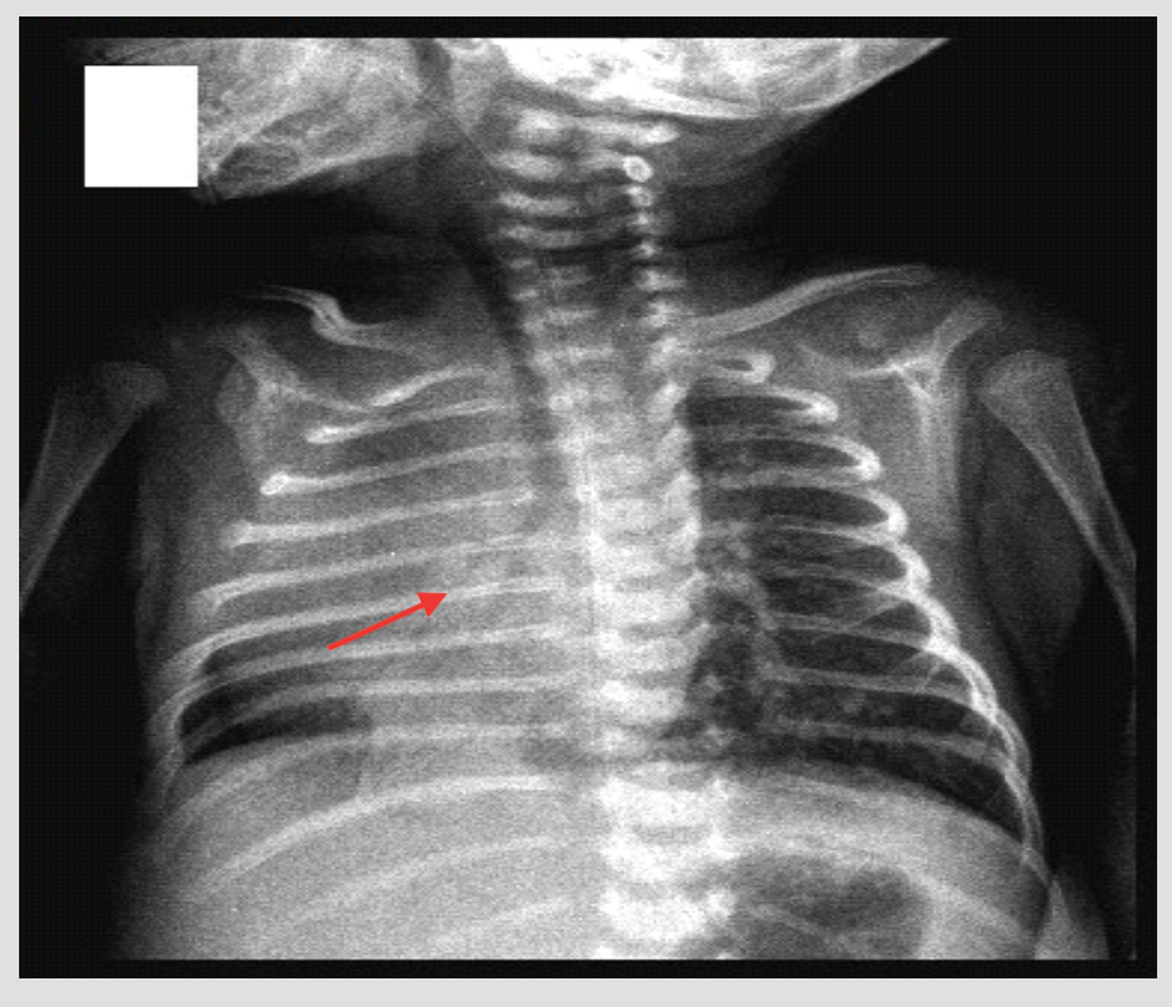
Chest x-ray of a 15 days old boy with Scimitar syndrome shows dextroposition of the heart.

Laboratory findings were as follows: hemoglobin 12.g/dl; hematocrit 32.5%; WBC 13000/mm^3^ (neutrophils 42%, lymphocytes 55%, and monocytes 3%); platelets 350,000/mm^3^; and C-reactive protein: negative. Other serum biochemical parameters were within normal limits. The initial chest x-ray suggested a lung parenchymal consolidation in the right mid zone and dextroposition of the heart (Figure 1).

**Figure 2:**
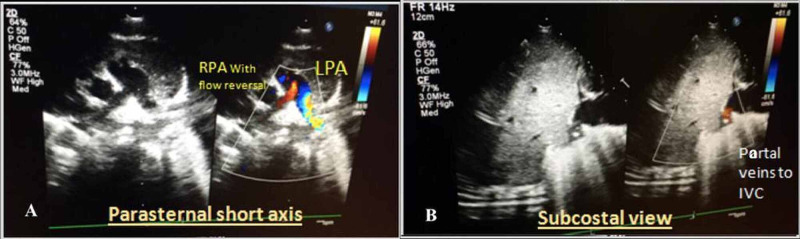
Echocardiography Figure 2A. Right pulmonary artery with flow reversal Figure 2B. Partial vein to inferior vena cava (IVC)

Echocardiography showed a mesocardium, dextroposition of the heart, abdominal situs solitus, atrial situs solitus, partial Rt veins to the inferior vena cava (IVC), an atrial septal defect (ASD II) (5mm) left-to-right shunt, moderately dilated right atrium (RA), right ventricle (RV) with trivial tricuspid regurgitation (TR) (37 mmHg), good biventricular systemic function, good size pulmonary artery (PA) with the retrograde flow at the right PA. No right ventricular outflow tract obstruction (RVOTO), no left ventricular outflow tract obstruction (LVOTO), no coarctation of the aorta (CoA), and no patent ductus arteriosus (PDA) were evident (Figure 2).

**Figure 3:**
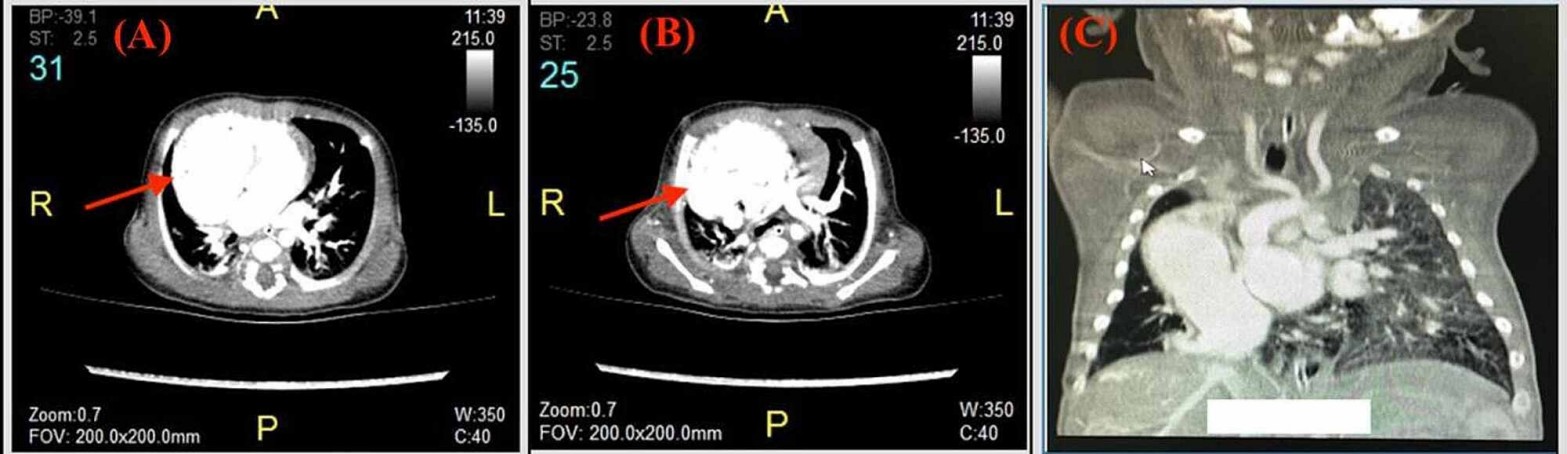
CT Chest (axial and coronal plane) Heart dextroposition and right pulmonary artery is smaller than left pulmonary artery

A CT scan of the chest was needed to confirm the diagnosis. It revealed right lower extralobar pulmonary sequestration, partial anomalous pulmonary venous drainage with right pulmonary parenchymal hypoplasia, pulmonary artery hypoplasia, and a pseudo horseshoe lung. Collateral was seen from abdominal aorta to the right lower lobe of the lung, the right pulmonary veins drained to the IVC before the entrance to the RA, and the left pulmonary veins to the LA indicated a dilated left lower pulmonary vein (Figure 3).

## Discussion

A 15-day-old male Saudi child presented to the ER with tachypnea and respiratory distress and was admitted to the nursery as a case of chest infection. He was later determined to have dextroposition of the heart, abdominal situs solitus, atrial situs solitus, partial right veins to the IVC, an ASD II (5mm) left-to-right shunt, moderately dilated RA and RV with trivial TR (37 mmHg) on echocardiography. We consider several differential diagnoses according to the findings in our case, and scimitar syndrome was one of the possible diagnoses that should not be ignored, despite its rare incidence of occurrence.

 The patient presented with tachypnea and respiratory distress, along with a chest infection, at the age of 15 days. Respiratory distress syndrome is one of the most common etiologies in patients with this presentation. Others include transient tachypnea of newborn and meconium aspiration syndrome. However, given our patient's history and findings, who was the product of normal vaginal delivery and full-term, and his presentation at 15 days of age with respiratory distress, we were alerted to search for other possible diagnoses.

 Excluding scimitar syndrome due to its low prevalence and rare incidence might lead to misdiagnosis as other differentials in many cases. A patient presenting with respiratory distress, tachypnea, recurrent chest infection, and failure to thrive, along with unusual chest x-ray findings, suggested that we investigate the possibility of scimitar syndrome. Therefore, we considered echocardiography and CT or MRI to check for possible findings of scimitar syndrome, such as dextroposition of the heart, abdominal situs solitus, atrial situs solitus, or partial Rt veins to the IVC on echocardiography. Our patient's CT scans confirmed right lower extralobar pulmonary sequestration, partial anomalous pulmonary venous drainage with right pulmonary parenchymal hypoplasia, pulmonary artery hypoplasia, and pseudo horseshoe lung on CT of the chest.

 This case report emphasizes the significance of considering scimitar syndrome in infants presenting with respiratory distress and the importance of doing a full workup before excluding this diagnosis. In different parts of the world, several cases reported in infants and adults have been published showing similar clinical presentations and investigation findings. For example, one case report documented a 3-day-old neonate boy who presented with a chest x-ray that revealed a curved shadow on the right middle lung zone extending to the diaphragm and abutting indenting the inferior vena cava (the scimitar sign). Transthoracic echocardiography and computerized tomographic angiography confirmed the presence of a scimitar vein, an associated dextroposition of the heart, and a hypoplastic right lung, along with other findings associated with scimitar syndrome; that patient also had an imperforate anus [10].

 Another case report described a 38-year-old adult woman who presented with dyspnea and fatigue for one week. Her chest x-ray showed dextrocardia and a right lung mass measuring 5×4cm. A CT scan of the chest revealed an accessory lobe of the liver extending into the chest cavity through a defect in the posterior right hemidiaphragm. It also showed the right pulmonary vein draining into the infra diaphragmatic inferior vena cava (IVC) instead of the left atrium, consistent with scimitar syndrome [11].

 A further case report presented a newborn Saudi male antenatally diagnosed with congenital hydrocephalus who developed mild respiratory distress soon after delivery. On examination, he showed markedly decreased breath sounds on the right side of the chest, with loud heart sounds in the pulmonary area. A chest x-ray, chest CT, ECG, and echocardiography confirmed the diagnosis of scimitar syndrome. The patient was treated with oxygen and antibiotics and transferred to a tertiary centre for catheterization to coil the collateral supplying the sequestrated lobe and later repair the partial vein [12].

## Conclusions

Our patient is considered one of the rare scimitar syndrome cases. The cardiologist was correct in his suspicions, and the requested radiological investigation confirmed his early diagnosis. Therefore, other tests should be considered to identify potential scimitar syndrome in patients presenting with respiratory distress and pulmonary infection. Advanced imaging techniques are sometimes needed to confirm the diagnosis.
